# An Improved Protocol for the Matrigel Duplex Assay: A Method to Measure Retinal Angiogenesis

**DOI:** 10.21769/BioProtoc.4899

**Published:** 2023-12-05

**Authors:** Kathleen C. Brown, Reagan S. Light, Kushal J. Modi, Kaitlyn B. Conely, Amanda M. Sugrue, Ashley J. Cox, Sarah L. Miles, Monica A. Valentovic, Piyali Dasgupta

**Affiliations:** Department of Biomedical Sciences, Joan C. Edwards School of Medicine, Marshall University, Huntington, WV, 25755 USA

**Keywords:** Angiogenesis, MDA, HRMEC, PP2, Quantification

## Abstract

Neovascular diseases of the retina, such as diabetic retinopathy (DR) and age-related macular degeneration (AMD), are proliferative retinopathies involving the growth of new blood vessels on the retina, which in turn causes impairment and potential loss of vision. A drawback of conventional angiogenesis assays is that they are not representative of the angiogenic processes in the retina. In the retina, the new blood vessels grow (from pre-existing blood vessels) and migrate into a non-perfused region of the eye including the inner limiting membrane of the retina and the vitreous, both of which contribute to vision loss. The Matrigel Duplex Assay (MDA) measures the migration of angiogenic capillaries from a primary Matrigel layer to a secondary Matrigel layer, which resembles the pathological angiogenesis in AMD and DR. The methodology of MDA is comprised of two steps. In the first step, the human retinal microvascular endothelial cells (HRMECs) are mixed with phenol red–containing Matrigel (in a 1:1 ratio) and seeded in the center of an 8-well chamber slide. After 24 h, a second layer of phenol red–free Matrigel is overlaid over the first layer. Over the course of the next 24 h, the HRMECs invade from the primary Matrigel layer to the secondary layer. Subsequently, the angiogenic sprouts are visualized by brightfield phase contrast microscopy and quantified by ImageJ software. The present manuscript measures the angiogenesis-inhibitory activity of the Src kinase inhibitor PP2 in primary HRMECs using the MDA. The MDA may be used for multiple applications like screening anti-angiogenic drugs, measuring the pro-angiogenic activity of growth factors, and elucidating signaling pathways underlying retinal angiogenesis in normal and disease states.

**Graphical overview**


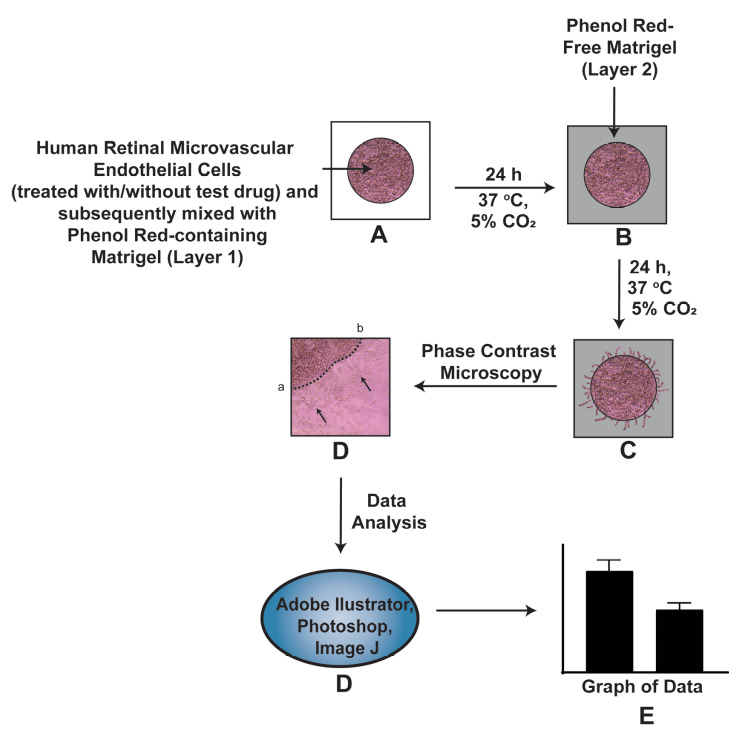


**Schematic representation of the Matrigel Duplex Assay (MDA) [7] set up in 8-well chamber slides.** (A) The first layer consists of human retinal microvascular endothelial cells (HRMECs) mixed in a 1:1 suspension with phenol red–containing Matrigel (PR-Matrigel, represented as Layer 1 in the figure above). The chamber slides (containing the primary layer) are incubated in a humidified tissue culture incubator maintained at 37 °C with 5% CO_2_ for 1 h. Subsequently, 250 µL of EGM-2 complete media is added to each well. The chamber slides are returned to the humidified tissue culture incubator (maintained at 37 °C with 5% CO_2_) and incubated for 24 h. After 24 h, the retinal endothelial cells grow and extend out to the boundary of this first layer. (B) A second layer of 1:1 solution of phenol red–free Matrigel (PR-free-Matrigel) in EGM-2 media (represented as Layer 2 in the figure) is overlaid on top of the first Matrigel layer. The cells are incubated for 1 h in a humidified tissue culture incubator maintained at 37 °C with 5% CO_2_. After 1 h, 250 µL of EGM-2 complete media is added to each well. The chamber slides are returned to the humidified tissue culture incubator (maintained at 37 °C with 5% CO_2_) and incubated for 24 h. (C) During these 24 h, the HRMECs invade (from the primary layer) into the secondary Matrigel layer. The chamber slides are visualized by phase contrast microscopy. (D) A representative photograph of the image obtained by the MDA is depicted. The black arrow indicates the HRMECs invading into the second layer of Matrigel. Using the Adobe Illustrator software, the dotted line (ab) is drawn at the interface between the two layers. The distance to which the cells have migrated (into the secondary Matrigel layer) is measured at six sites within each photograph in a randomized double-blind fashion by three independent observers, using NIH ImageJ Version 1.47. This process is repeated for three separate photographic fields per sample. (E) These measurements were used to draw the graph of the data using GraphPad Prism.

## Background

The retina consists of organized layers of photoreceptors, interneurons, glia, epithelial cells, and endothelial cells. The proper maintenance of vascular networks is critical to normal visual function [1]. Aberrant angiogenesis is the hallmark of several ocular diseases including age-related macular degeneration (AMD) and diabetic retinopathy (DR) [2]. The angiogenic process in the retina is a complex, multistep process involving endothelial cell invasion, adhesion, chemotactic migration, proliferation, and differentiation into capillary tube–like structures and the production of a basement membrane around the vessel [3]. A survey of literature shows that the Matrigel capillary tube assay is one of the most prevalent angiogenesis assays in cell culture models [4, 5]. In this assay, human microvascular endothelial cells are seeded on a three-dimensional layer of solidified Matrigel (or any other reconstituted basement membrane extracellular matrix). Over a period of 24 h, the cells differentiate into capillary tube–like networks, which can be quantified by digital image analysis. A caveat of this assay is that it does not represent pathological angiogenesis in the eye. The Matrigel assay is more representative of vasculogenesis, which is defined as the differentiation of endothelial cells to yield de novo primitive vascular networks rather than angiogenesis where new capillaries are generated from existing vasculature [6]. Another disadvantage is the lack of a lumen in the capillaries obtained by this assay [7, 8]. Other methods used to measure retinal angiogenesis include the measurement of endothelial cell proliferation or endothelial cell chemotaxis or migration. Although these individual processes are useful indicators of angiogenic activity, they do not provide a holistic representation of the angiogenic cascade [7, 8]. These drawbacks are circumvented by using the Matrigel Duplex Assay (MDA). In the MDA, the angiogenic sprouting (in the secondary Matrigel layer) arises from pre-formed vascular networks in the primary layer [9, 10].

The MDA represents a combination of all the steps of retinal angiogenesis and provides a highly relevant model for the study of pro- and anti-angiogenic agents in vitro. In-depth morphological studies have shown that the angiogenic sprouting (occurring in the MDA) closely mimics retinal angiogenesis in vivo. Electron microscopy studies have demonstrated the presence of a lumen and elaborate cell–cell junctions within the endothelial cell aggregates observed in the MDA [9, 10]. As the endothelial capillary tube–like structures invade into the secondary Matrigel layer, the leading edge of the capillary sprouts is associated with long filopodia. The structure of these filopodia resembles those seen at the angiogenic front during developmental angiogenesis in the neonatal retina [9]. The retinal endothelial cells grown within the two layers of Matrigel reflect the biological characteristics of the neovascular retina, such as diffusion gradient of oxygen, nutrients, and pH. The growth of the cells inside the Matrigel duplex system allows for complex cell–cell and cell–matrix interaction. Furthermore, these retinal endothelial sprouts may be characterized and quantified by confocal microscopy [9].

Stitt et al. (2005) were the first to report the protocol for the MDA to study retinal angiogenesis in diabetic retinopathy [10]. During the application of the original protocol of Stitt et al. (2005) in our laboratory, some technical challenges occurred. The original protocol uses phenol red–containing Matrigel for both the primary and the secondary Matrigel layers [9, 10]. We observed that the interface between the two layers was difficult to visualize by phase contrast microscopy. This is especially true when there is a dense network of retinal capillaries migrating from the primary to the secondary Matrigel layer in the MDA. In order to circumvent this issue, we used a black Sharpie marker pen (in our early studies) to draw a boundary encircling the first layer before adding the second Matrigel layer [11]. This approach had its limitations as it was difficult to accurately demarcate the boundary of the first layer. Secondly, the black boundary around the primary layer appeared as a thick black band under the brightfield microscope, increasing the difficulty of accurately visualizing and quantifying the angiogenic capillary sprouts in the second layer. We drew the dotted line in the center of this black band to quantify the invasion of endothelial capillary networks into the secondary layer [11] and the whole process became arduous and cumbersome.

We devised a novel strategy to clearly differentiate between the two Matrigel layers in the MDA by utilizing phenol red–free Matrigel (PR-free-Matrigel) and phenol red–containing Matrigel (PR-Matrigel) for the individual layers. Initially, we tried to use PR-free-Matrigel for the primary layer and PR-Matrigel for the secondary layer but found that this arrangement resulted in poor quality photographs under a microscope. Subsequently, we reversed the layers (using the PR-Matrigel in the primary layer and PR-free-Matrigel in the secondary layer) and obtained clearer pictures under phase contrast microscopy, providing readily reproducible images and assay results. The present manuscript describes the ability of the Src Kinase inhibitor PP2 [12] (at a concentration of 10 µM) to suppress retinal angiogenesis of human retinal microvascular endothelial cells (HRMECs) using our modified MDA protocol. We hope that the MDA will be a useful tool for researchers working in the field of physiological and pathological angiogenesis in the retina.

## Materials and reagents

1. Primary human retinal microvascular endothelial cells (HRMECs) (Cell Systems, catalog number: ACBRI 181). Grow HRMECs in endothelial growth media-2 (EGM-2) [EGM^TM^-2 Endothelial Cell Growth Medium-2 BulletKit^TM^ (Lonza, catalog number: CC-3162)]. The BulletKit^TM^ contains EBM-2 basal medium (catalog number: CC-3156) and SingleQuots growth factor supplements (catalog number: CC-4176) needed for the growth of HRMECs. The BulletKit^TM^ also contains a vial of antibiotic (Gentamycin) and fetal bovine serum (FBS). Store the EBM-2 at 4 °C and the SingleQuots and FBS at -20 °C. Prepare the EGM-2 media following the manufacturer’s instructions provided in the BulletKit^TM^. Add FBS to the media at a final concentration of 2% (see Recipes). The EGM-2 medium with 2% FBS will be hereafter referred to as EGM-2 complete medium
2. RPMI-1640 medium (ATCC, catalog number 30-2001)
3. Trypsin-EDTA (0.25% Trypsin, 0.53 mM EDTA) (ATCC, catalog number: 30-2101). Long-term storage at -20 °C; however, aliquot Trypsin-EDTA and store at 4 °C for immediate use
4. Dulbecco’s Phosphate buffered saline (DPBS) without calcium and magnesium (Corning, Fisher, catalog number: 21-031-CM). Store at 4 °C
5. Absolute anhydrous ethanol (200 proof) (Pharmaco, Greenfield Global, catalog number: 111000200). Dilute ethanol with distilled water to obtain a 70% ethanol solution for wiping down the laminar flow hood
6. PR-Matrigel membrane matrix (Fisher, catalog number: CB-40234). Aliquot the Matrigel in Corning sterile freezing vials and store at -70 °C for long-term storage. Before the experiment, keep the Matrigel aliquot overnight at 4 °C (in a refrigerator) for thawing. During the assay, Matrigel should be kept on ice throughout the experiment
7. PR-free-Matrigel membrane matrix (Fisher, catalog number: CB-40234C)
8. PP2 (Enzo Biosciences, catalog number: 50-201-0681). PP2 is a potent and selective inhibitor of the Src family tyrosine kinases). Store PP2 at -20 °C and solubilize in DMSO (Fisher, catalog number: MT-25950CQC) to yield the stock solution at concentration of 10 mM (see Recipes). Aliquot the PP2 stock solution into microcentrifuge tubes and store at -20 °C. Immediately before use, thaw and dilute the PP2 in EBM-2 basal media (without FBS or growth factors) to a working concentration of 10 µM. After use, discard the remaining solution of 10 µM PP2

**Tissue culture plasticware and related supplies**

1. Corning cell counting chamber (Fisher, catalog number: 07-200-988)
2. Nunc Lab-Tek 8-well Permanox plastic chamber slide system (Fisher, catalog number: 12-565-22)
3. Corning BioCoat Collagen-coated T-25 (Fisher, catalog number: 08-774-327) and T-75 (Fisher, catalog number: 08-774-327) tissue culture flask. The BioCoat flasks are stored at 4 °C
4. Corning sterile cryogenic vials (Fisher, catalog number: 13-700-504)
5. Sterile polypropylene centrifuge tubes, 50 mL (Fisher, catalog number: 06-443-20)
6. Sterile polypropylene centrifuge tubes, 15 mL (Fisher, catalog number: 05-539-5)
7. Safety vented wash bottles (1,000 mL) for storing 70% ethanol (Fisher, catalog number: 11-865-170)
8. Microcentrifuge tubes, 1.5 mL (Fisher, catalog number: 05-408-129). Autoclave the microcentrifuge tubes before using them for the MDA
9. Sterile 5 mL microcentrifuge tube (Eppendorf, catalog number: 0030119487)
10. Sterile plastic serological pipettes (individually Wrapped, paper peel packaging, plugged):
  25 mL pipette (Fisher, catalog number: 12-567-604)
  10 mL pipette (Fisher, catalog number: 12-567-603)
  5 mL pipette (Fisher, catalog number: 12-567-602)
  2 mL pipette (Fisher, catalog number: 12-567-601)
  1 mL pipette (Fisher, catalog number: 12-567-600)
11. Fisherbrand 5 3/4 inches’ disposable borosilicate glass Pasteur pipettes (Fisher, catalog number: 13-678-20B). Autoclave the Pasteur pipettes before using them for the MDA
12. Fisherbrand sterile high precision #22 style scalpel blade (Fisher, catalog number: 12-000-161)
13. Drummond^TM^ Portable Pipet-Aid^TM^ XP pipette Controller (Fisher, catalog number: 13-681-15E)
14. PIPETMAN^®^ 4-Pipette kit P2, P20, P200, P1000 (Gilson, catalog number: F167360)
15. Fisherbrand SureOne sterile aerosol barrier tips (100–1,000 µL) (Fisher, catalog number: 02-707-404)
16. Fisherbrand SureOne sterile aerosol barrier tips (20–200 µL) (Fisher, catalog number: 02-707-430)
17. Fisherbrand SureOne sterile aerosol barrier tips (0.1–20 µL) (Fisher, catalog number: 02-707-470)

**Other consumable laboratory supplies**

1. Labcoat (Fisher, catalog number: 19-181-596)
2. Ansell MICROFLEX^TM^ NeoPro^TM^ NEC-288 Neoprene small size (Fisher, catalog number: 19-350-340F)
  and medium size gloves (Fisher, catalog number: 19-350-340A)
3. Freezer boxes for long-term storage of Matrigel and PP2 (Cole-Parmer Essentials 81-Place Freezer Boxes, catalog number: UX-04396-63)
4. Kimtech Kimwipe delicate task wipers (Fisher, catalog number: 06-666A)
5. Sterile nonwoven gauze sponges 4 × 4 inch (Fisher, catalog number: 22-028-558)
6. Hayman style microspatula (Fisher, catalog number: 21-401-25A)

**Solutions**

1. EGM-2 complete medium (see Recipes)
2. EGM-2 media (with growth factors) (see Recipes)
3. 10 mM PP2 (see Recipes)
4. 200 µM PP2 (see Recipes)
5. VEH-20X (see Recipes)

**Recipes**

1. **EGM-2 complete medium**
  The EGM^TM^-2 Endothelial Cell Growth Medium-2 BulletKit^TM^ contains the following ingredients:
  1× EBM-2 basal medium (CC-3156), 500 mL
  1× EGM-2 SingleQuots supplement pack (CC-4176) containing:
  - 1× white cap vial with VEGF, 0.50 mL
  - 1× gray cap vial with hFGF-B, 2 mL
  - 1× yellow cap vial with R3-IGF-1, 0.50 mL
  - 1× green cap vial with hEGF, 0.50 mL
  - 1× orange cap vial with heparin, 0.50 mL
  - 1× blue cap vial with ascorbic acid, 0.50 mL
  - 1× red cap vial with GA-1000, 0.50 mL
  - 1× natural cap vial with hydrocortisone, 0.20 mL
  - 1× bottle FBS, 10 mL
    Please use the following table to prepare the required volume of EGM-2 complete medium:
    **Table:** 

EGM-2 complete media (mL)	VEGF (µL)	FGF (µL)	IGF-1 (µL)	EGF (µL)	Heparin (µL)	Ascorbic acid (µL)	Gentamycin (GA-1000) (µL)	Hydrocortisone (µL)	FBS (mL)
50	50	200	50	50	50	50	50	20	1
100	100	400	100	100	100	100	100	40	2
250	250	1,000	250	250	250	250	250	100	5
400	400	1,600	400	400	400	400	400	160	8
500	500	2,000	500	500	500	500	500	200	10
2. **EGM-2 media (with growth factors)**
  We usually make EGM-2 media (with growth factors) in a bottle and add the FBS to the T-75 flask directly. This is because we often do experiments in serum-free conditions, so we add the FBS separately during cell culture as required.
3. **10 mM PP2**
  Molecular weight of PP2 = 301.78
  Weigh 6 mg of PP2 in a sterile microcentrifuge tube. In the laminar flow hood, dissolve the PP2 in 2 mL of DMSO. Vortex briefly to obtain 10 mM PP2 stock solution. Aliquot this PP2 stock solution (as 50 µL aliquots) into microcentrifuge tubes and store at -20 °C.
4. **200 µM PP2**
  Thaw out one aliquot of PP2 (concentration = 10 mM) in a water bath (held at 37 °C). Add 2 µL of PP2 in 100 µL of basal EBM-2 medium. Vortex vigorously. Now the concentration of PP2 is 200 µM (called PP2-20X in the text). Use this PP2 solution as described in Section D, Step 8. Discard the remaining solution of PP2-20X.
5. **VEH-20X**
  Add 2 µL of DMSO in 100 µL of basal EBM-2 medium. Vortex vigorously to obtain VEH-20X solution (the concentration of DMSO in this solution is 2% v/v), which is referred to as VEH-20X. Use the VEH-20X solution as described in Section D, Step 10. Discard the remaining solution of VEH-20X.

## Equipment

1. NU-540 (LabGard^®^ ES NU-540 class II, type A2) laminar-flow biosafety cabinet (NuAire, Plymouth, MN, catalog number: NU-540)
2. Cell culture incubator (Heracell VIOS 150i cell culture incubator) (Thermo Scientific, Waltham, MA, catalog number: 51-032-872)
3. Leica DM IL LED inverted phase contrast microscope with camera (VWR International, catalog number: 76382-982)
4. Fisher Isotemp 220A water bath (Fisher, catalog number: FSGPD05)
5. Thermo Electron Corporation IEC Centra CL2 benchtop centrifuge (Thermo Scientific, catalog number: 004260F)
6. Microbalance (Sartorius, Model 1712 MP8, silver edition, catalog number: CUBIS_II_ANALYTICAL)
7. Vortex mixer (Fisher, catalog number: 02-215-414)

## Software

1. LAS Image Capture Software (Leica Microsystems)
2. Adobe Photoshop 2023 (Adobe Creative Cloud for Windows)
3. Adobe Illustrator 2023 (Adobe Creative Cloud for Windows)
4. NIH ImageJ Version 1.47 (National Institutes of Health, Bethesda)
5. GraphPad Prism Version 9

## Procedure

- **Culturing HRMECs: thawing HRMECs from freezing vial**
  1. Order cryopreserved HRMECs from Cell Systems. These HRMECs are isolated from normal, healthy donor tissue and supplied at passage 3 (<12 cumulative population doublings). A certificate of analysis is provided with each vial, which contains approximately 1 × 10^6^ cells. After obtaining the cryopreserved cells from the vendor, they should be immediately stored in liquid nitrogen. We usually thaw the HRMECs by adding one frozen cell vial into one BioCoat T-25 and one T-75 flask. All steps are done in a laminar flow hood under aseptic conditions.
  2. Rehydration of BioCoat Collagen-coated T-25 and T-75 tissue culture flasks:
    The BioCoat flasks are stored at 4 °C. Take the packet containing the flasks out and allow them to come to room temperature prior to use. Warm some bicarbonate-based serum-free culture medium (such as RPMI-1640) in a 37 °C water bath. Add 5 mL of warm bicarbonate-based serum-free culture medium (such as RPMI) to the T-25 flask. Similarly, add 15 mL of warm serum-free media to the T-75 flask. We usually use warm RPMI-1640 for rehydrating the flasks. Allow the flasks to rehydrate for 2 h in a humidified tissue culture incubator maintained at 37 °C with 5% CO_2_. After 2 h, aspirate the media from the flask, taking care not to disrupt the collagen coating.
  3. Add 10 mL of EGM-2 complete media to the rehydrated T-25 flask. Similarly, add 20 mL of EGM-2 complete media to the rehydrated T-75 flask. **Important:** Cryopreserved cells are very delicate. The HRMECs are typically cryopreserved in complete EGM-2 media supplemented with 5% DMSO. Thaw the vial in a 37 °C water bath by swirling the vial gently until the contents are completely thawed. Each vial contains approximately 1 × 10^6^ cells at passage 3 (<12 cumulative population doublings).
  4. As soon as the vial is thawed, immediately remove the vial from the water bath, wipe it dry, and rinse it with 70% ethanol. Subsequently, transfer the vial inside a laminar flow hood. Remove the cap, being careful not to touch the interior threads with gloved fingers.
  5. Using a 1 mL sterile serological pipette, gently dispense the contents of the vial into the rehydrated BioCoat T-25 and T-75 flasks containing EGM-2 complete media (Figure 1) at the recommended seeding density of 6,000 cells/cm^2^. Rinse the freezing vial with 1 mL of EGM-2 complete medium and add it to the tissue culture flasks. **Important:** Do not centrifuge the cells from the freezing vial. The DMSO present in the cryopreserved cells will get diluted in the EGM-2 complete media present in the flask. Therefore, the cells will not be harmed by the DMSO.
    **Figure 1.**
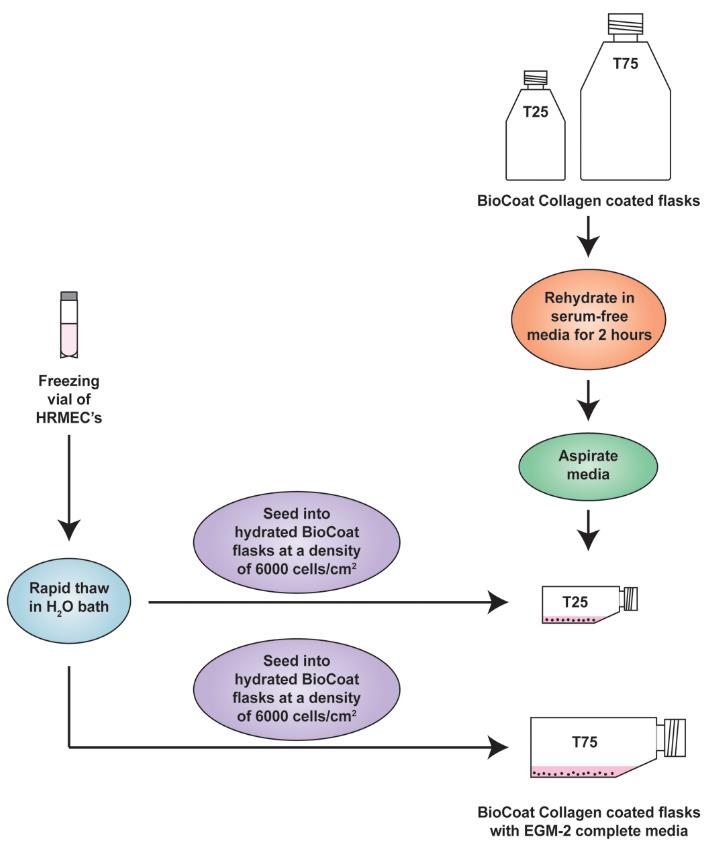
Schematic diagram representing the establishment of human retinal microvascular endothelial cell (HRMEC) cultures from cryopreserved cells. The flasks should be placed flat (horizontally) in the cell culture incubator.
  6. Return the tissue culture flasks to the humidified tissue culture incubator maintained at 37 °C with 5% CO_2_. Do not disturb the culture for at least 24 h after the culture has been initiated. After 24 h, aspirate and discard the media in the flask and add fresh EGM-2 complete media to remove any residual DMSO and unattached cells. A healthy culture will display cobblestone morphology and non‐granular cytoplasm [9, 10].
- **Culturing HRMECs: maintenance and subculturing of HRMEC cultures**
  1. Culture HRMECs to approximately 70%–80% confluence. The media should be replenished once every three days.
  2. Rehydrate the required number of BioCoat T-75 flasks (see Section A, Step 2).
  3. Rinse the cells with DPBS. Use 5 mL of DPBS for T-25 and 10 mL of DPBS for T-75 flasks.
  4. Add 3 mL of 0.25% Trypsin/EDTA solution into the T-75 flask (in the case of T‐25 flask, use 2 mL).
  5. Gently rock the flask to ensure that the cells are covered by Trypsin/EDTA solution. Incubate the flask at 37 °C incubator for 1–2 min or until cells are completely rounded up (monitored with inverted microscope). Gently tap the side of the flask to fully detach the cells if necessary.
  6. Add 2 mL of FBS to the T-75 flask (use 1 mL for a T-25 flask) to neutralize the Trypsin-EDTA. Using a 5 mL serological pipette, gently rinse the flask completely and transfer the cells in a 15 mL centrifuge tube.
  7. Rinse the growth surface of the flask with 10 mL of DPBS to collect the remainder of the detached cells and combine in the same 15 mL centrifuge tube from Step B6. Close and examine the flask under an inverted microscope to verify that the cell harvesting is complete. There should be less than 5% cells (of the initial density of the flask) remaining in the flask.
  8. Centrifuge the harvested cell suspension at 800× *g* for 5 min at room temperature using a benchtop centrifuge. Remove (aspirate) the FBS/DPBS liquid from the pellet. Gently flick the tube to dislodge the cells and resuspend them in 1 mL of EGM-2 complete medium.
  9. Count the cells using a hemocytometer and then dispense the required volume of cell suspension into the hydrated T-75 flask as recommended in see Section A, Steps 5–6. Return the flasks back to the cell culture incubator maintained at 37 °C and 5% CO_2_.
- **Working with Matrigel**
  1. The Matrigel is aliquoted in ice-cold sterile Corning freezing vials and stored at -70 °C.
  2. Before starting the MDA, the required amount of Matrigel should be thawed overnight at 4 °C. The Matrigel is always kept on ice during the experiment.
  3. Matrigel is a liquid at 4 °C, and it polymerizes into a gel-like solid at 37 °C. We recommend that plasticware and reagents should be kept ice cold while handling the Matrigel.
  4. The thawed Matrigel is a thick, viscous liquid. Pipette tips should be trimmed at the tip (to increase the diameter of the tip) to transport the Matrigel in/out of the freezing vials. All microcentrifuge tubes and pipette tips should be kept ice cold while handling the Matrigel to avoid polymerization. In our laboratory, we use ice-cold serum-free RPMI-1640 as a cooling media. All pipette tips are dipped into the ice-cool media to chill them down before using them to handle Matrigel.
- **Matrigel Duplex Assay**
  **Day 1**
  1. The assay is set up in an 8-well chamber slide. Before starting the experiment, draw the schema of the assay (Figure 2). The region (with the checkered pattern) of the right side of the chamber slide is used to lift and transport the slide.
    **Figure 2.**
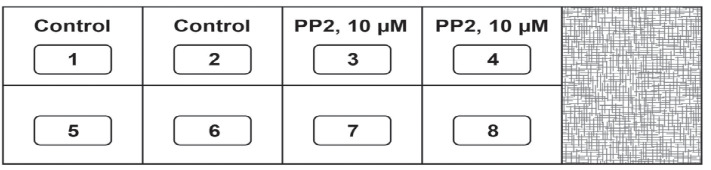
Schema of the Matrigel Duplex Assay
  2. All experiments are performed with HRMECs between passage 3–7. The entire experiment is performed in a laminar flow hood under aseptic conditions.
  3. Grow the HRMECs cells to approximately 70%–80% confluence (in hydrated collagen-coated BioCoat T-75 flasks (please see Section A, Step 2 on instructions for rehydration of flasks) in complete EGM-2 media in a humidified environment at 37 °C (with 5% CO_2_) in a cell culture incubator.
  4. Wash the HRMECs once with DPBS and trypsinize the flask using 0.25% Trypsin-EDTA.
  5. Add 2 mL of FBS to neutralize the trypsin and transfer the cell suspension to a 15 mL tube. Rinse the flask with DPBS and transfer the solution to a 15 mL tube.
  6. Gently spin down the cells at 800× *g* for 5 min at room temperature in a benchtop centrifuge. Aspirate the media and resuspend the HRMECs in EGM-2 containing 4% FBS.
  7. Count the cells using the Corning cell counting chamber. Adjust the concentration of the cells to 1.6 × 10^7^ cells/mL (using EGM-2 media containing 4% FBS) in a sterile 5 mL microfuge tube. This tube is labeled as **TUBE A.**
  8. The stock solution of PP2 (10 mM) should be diluted to a concentration of 200 µM (see Recipes) in basal EBM-2 media in a sterile microfuge tube. The PP2 is prepared at 20× working concentration and labeled as PP2-20X. The PP2-20X is the concentration of PP2 used to set up the MDA. The final concentration of PP2 to be used for the MDA is 10 µM.
  9. In a separate 1.5 mL microfuge tube, mix the cells (from **TUBE A**) with PP2-20X at the ratio of 10:1 (v/v). This dilutes the PP2-20X to a concentration of 20 µM. As an example, you may add 45 µL of cell suspension (from **TUBE A**) to 5 µL of the PP2-20X. Gently flick the tube so that the drug mixes well with the cell suspension. Do not vortex because this will shear (and lyse) the cells. Therefore, the new concentration of PP2 is 20 µM. This tube is labeled as **TUBE B.**
  10. The vehicle for the PP2-20X is 2% DMSO (hereafter referred to as VEH-20X, see Recipes). Just like in the previous step, mix the cells (from **TUBE A**) with VEH-20X at the ratio of 10:1 (v/v). Do not vortex because this will shear (and lyse) the cells. Therefore, the new concentration of the vehicle is now 0.2% DMSO. This tube is labeled as **TUBE B-VEH.**
  11. Keep a few empty 1.5 mL microfuge tubes on ice to chill them down. Combine the cells with Matrigel at a ratio of 1:1 in an ice-cold microfuge tube. For example, mix 20 µL of Matrigel with 20 µL of cell suspension (taken from **TUBE B**). Mix the cells with the Matrigel by flicking the tube. Do not allow the tube to warm up and do not generate air bubbles in the Matrigel–cell suspension while flicking. The final PP2 concentration in the tube is now 10 µM. This tube is labeled as **TUBE C**.
  12. Similarly, 20 µL of HRMECs (treated with 0.2% DMSO) are mixed with 20 µL of Matrigel in a separate microfuge tube. The final concentration of vehicle in the tube is now 0.1% DMSO. This tube is labeled as **TUBE C-VEH.**
  13. Table 1 describes the tubes A, B, and C in detail.
    **Table 1.** Description of tubes A, B, and C in the Matrigel Duplex Assay

Tube name	Composition of vehicle	Contents of tube
**A**		HRMECs resuspended in EGM-2 media supplemented with 4% FBS at a concentration of 1.6 × 10^7^ cells/mL.
**B**		45 µL of HRMEC suspension (a concentration of 1.6 × 10^7^ cells/ml, taken from **TUBE A**) mixed with 5 µL of 200 µM PP2 (referred in the text as PP2-20X). Final concentration of PP2 is now 20 µM. Gently flick the tube so that the drug mixes well with the cell suspension.
**B-VEH**	The vehicle for the PP2-20X is 2% DMSO (referred in the text as VEH-20X)	45 µL of HRMEC suspension (a concentration of 1.6 × 10^7^ cells/mL, taken from **TUBE A**) and mixed with 5 µL of 2% DMSO (which is the vehicle for PP2-20× and also called VEH-20% in the text).
**C**		Mixture of 20 µL of Matrigel with 20 µL of cell suspension (treated with PP2 and taken from **TUBE B**). Mix the cells with the Matrigel by flicking the tube.
**C-VEH**		**TUBE C-VEH** contains a mixture of 20 µL of Matrigel with 20 µL of cell suspension (treated with vehicle and taken from **TUBE B-VEH**). Mix the cells with the Matrigel by flicking the tube.
  14. Take a chamber slide out of the packet. Place the chamber slide in a dish over a piece of tissue paper inside the laminar flow hood. Use a Kimwipe or a sterile gauze pad to prevent the chamber slide from sticking to the surface of the hood, as this may cause a disruption of the primary Matrigel layer during the transfer to the incubator.
    Take 6 µL of the cell suspension from **TUBE C** and plate it as a drop in the center of the chamber slide. Each sample is assayed in duplicate. A similar process is followed for **TUBE C-VEH**. The chamber slide is then incubated at 37 °C for 1 h in a humidified cell culture incubator with 5% CO_2_. Below is a schematic representation of the chamber slide with the drop in the middle of the well (Figure 3). We also provide a photograph of the chamber slide with the drop in the middle of the well (Figure 4).
    **Figure 3.**
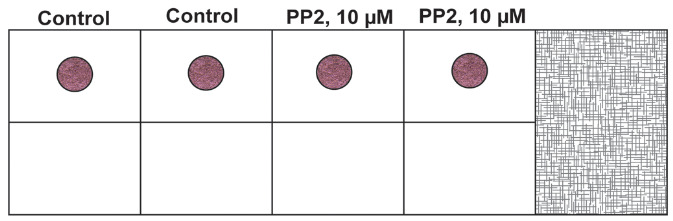
Schematic diagram of the chamber slide with the drop in the middle of the well
*Note: The drop does not need to be exactly at the geometric center of the slide. The important fact to remember is that it should be centered enough so that the cells can invade radially into the secondary layer. Even if the drop (the primary layer) is slightly off center, that would be okay for the assay.*
    **Figure 4.**
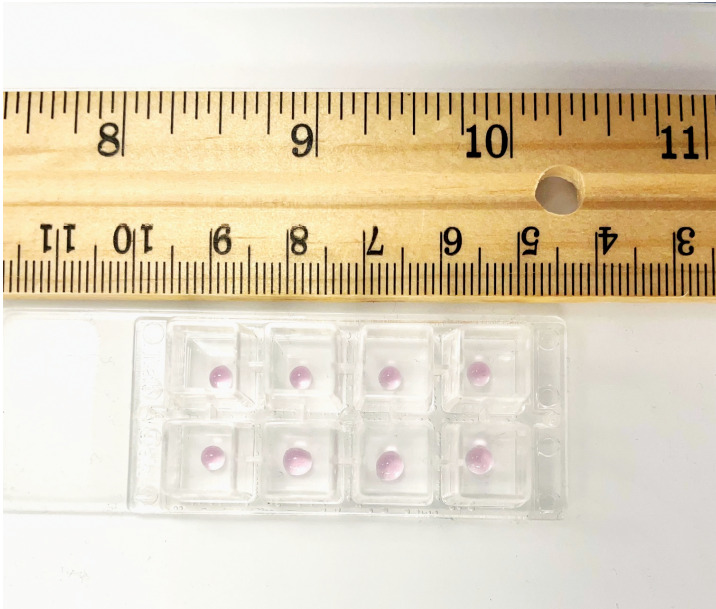
Photograph of the chamber slide with the primary layer in the middle of the slide
  15. After 1 h, add 250 µL of EGM-2 complete media to each well. Leave the chamber slide at 37 °C (with 5% CO_2_) for 24 h in a cell culture incubator.
  16. Thaw a vial of PR-free Matrigel overnight at 4 °C. If the assay is set up as shown in the schematic diagram above (Figure 5), 1 mL of PR-free Matrigel will be required for the experiment.
    **Figure 5.**
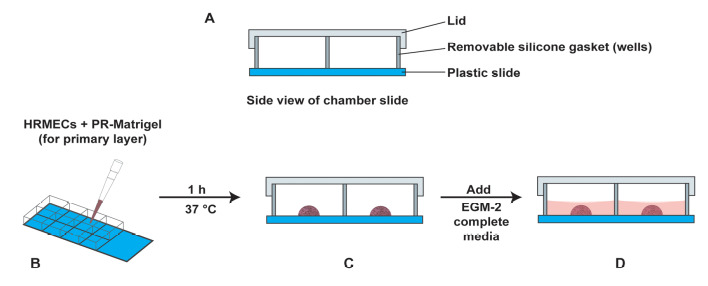
Schematic diagram of the side view of the chamber slide. (A) The components of the chamber slide have been labeled. (B) The procedure of adding the primary layer (PR-containing Matrigel and HRMECs) in the center of the chamber slide. (C) The drop is allowed to polymerize for 1 h at 37 °C (with 5% CO_2_) in a cell culture incubator. (D) After 1 h, 250 µL of EGM-2 complete media is added to each well.
**Day 2**
  1. After 24 h, dilute the PR-free Matrigel with an equal volume of EGM-2 supplemented with 4% FBS. Keep this solution on ice.
  2. Look at the chamber slide under a phase contrast microscope to confirm the presence of a network of retinal endothelial cell tube–like structures within the primary Matrigel layer (the drop).
  3. Aspirate the medium from the chamber slide. Use a pipette to gently remove the medium from the cell without disturbing the drop at the center of the chamber slide.
  4. Gently overlay 200 µL of the 1:1 solution of phenol red-free Matrigel (diluted in EGM-2 supplemented with 4% FBS) over the drop. Figure 6 represents a schematic diagram of the chamber slide. The pink color drop represents the primary layer of Matrigel, and the light grey region (around the pink drop) represents the secondary layer comprised of phenol red–free Matrigel. A side view of the chamber slide is provided in Figure 7. The chamber slide is incubated at 37 °C for 1 h (with 5% CO_2_) in a cell culture incubator. After 1 h, add 250 µL of EGM-2 complete media to each well. Leave the chamber slide at 37 °C for 24 h in a cell culture incubator.
    **Figure 6.**
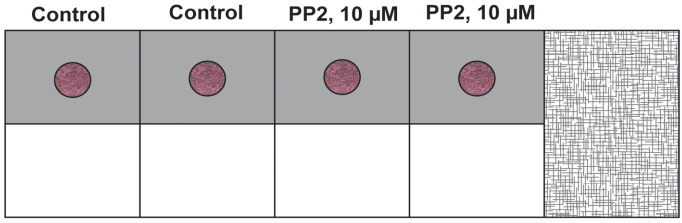
Schematic diagram of the chamber slide with both the primary and secondary layers
    **Figure 7.**
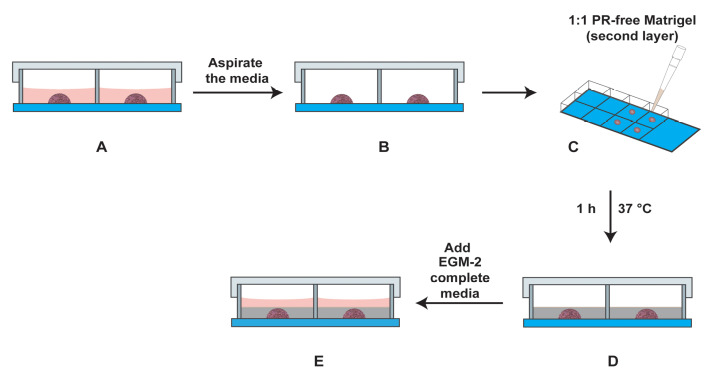
Schematic diagram of the side view of the chamber slide with the primary and secondary layers in the well. (A) Side view of chamber slide with the primary layer (drop in the center) covered with EGM-2 media (light red color). (B) The media is aspirated carefully. (C) The second layer comprising a 1:1 solution of EGM-2 media (containing 4% FBS) and PR-free Matrigel is overlaid on the primary layer. (D) The secondary layer (indicated by grey color) is allowed to polymerize for 1 h at 37 °C in a cell culture incubator. (E) Subsequently, EGM-2 media (indicated in light red color) is added to the well, and the slide is incubated at 37 °C for 24 h (with 5% CO_2_) in a cell culture incubator.
**Day 3**
  After 24 h, observe the chamber slide under a phase contrast microscope. The angiogenic tube–like structures (from the HRMECs in the primary Matrigel layer) should be clearly seen invading radially into the secondary Matrigel layer. Photograph three independent fields (at 10× magnification) for each sample for quantitative analysis.
- **Processing the images using Adobe Illustrator and ImageJ**
  1. The figure shown below shows the representative images obtained from the MDA. The black arrows indicate the sprouting angiogenic HRMEC tube–like structures, which have invaded the secondary Matrigel layer from the primary layer (Figure 8).
    **Figure 8.**
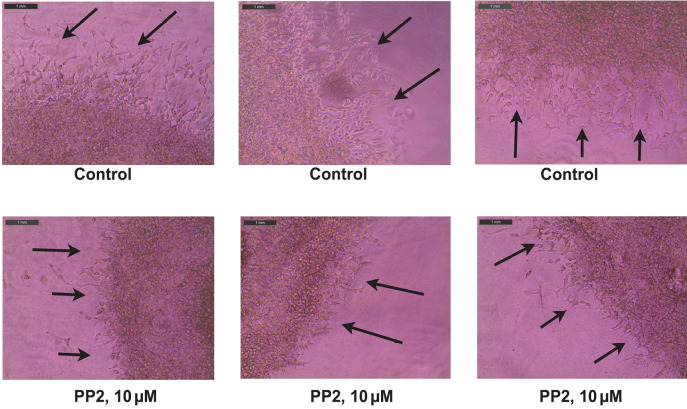
Representative photographs obtained from the Matrigel Duplex Assay (MDA). The black arrows indicate the angiogenic tube–like structures invading into the secondary layer.
  2. Open the images in Adobe Illustrator 2023. Using the pen curvature tool, draw a dotted line across the interface between the two layers. A representative image with the dotted lines is shown below (Figure 9).
    **Figure 9.**
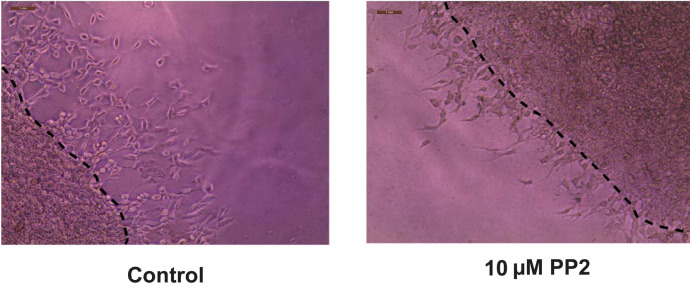
Drawing the dotted line at the interface of the two layers using Adobe Illustrator 2023
  3. Export the images to Adobe Photoshop 2023 and save them in jpeg format. Open the jpeg files in ImageJ. Using the line tool, draw a set of six lines marking the distance that the angiogenic tube–like structures have invaded into the secondary Matrigel layer (Figure 10). After drawing each line, press CTRL-M to obtain the length of the line drawn. Cease drawing the line upon encountering a gap larger than two cells. Our microscope images were analyzed using the ImageJ program by three independent observers in a double-blind manner. The raw data is provided in Supplementary information.
    **Figure 10.**
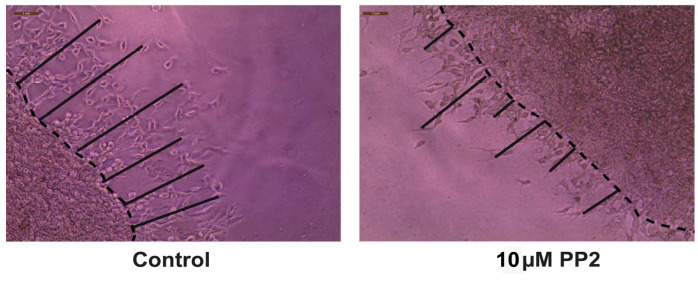
Representative image of the Matrigel Duplex Assay (MDA) with lines drawn using Adobe Illustrator
  4. Open GraphPad Prism (Version 9). Using the numbers obtained by the ImageJ program, create a column graph of the data and perform statistical analysis (Figure 11). The statistical analysis of the data is also described in the Data analysis section.
    **Figure 11.**
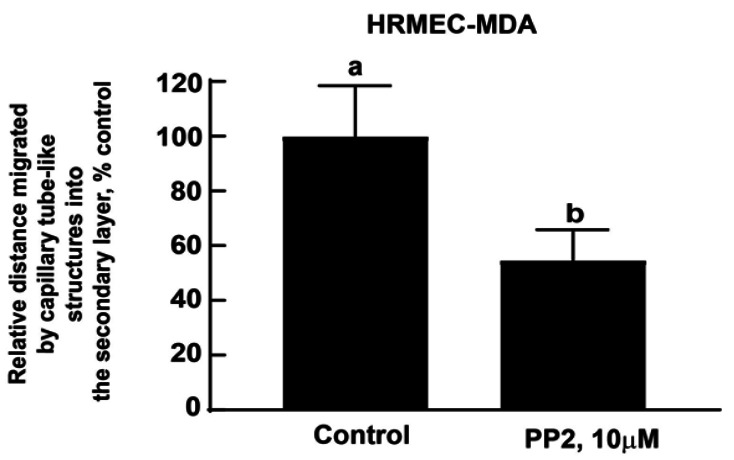
Microscope images were analyzed by the ImageJ program (by three independent observers). Data were graphically represented using GraphPad Prism (Version 9). Data were analyzed through an unpaired non-parametric *t*-test followed by the Mann-Whitney test. Values represented by the same letter are not statistically significantly different from each other (*P* ≤ 0.05).

## Data analysis

Each sample was tested in duplicate in the MDA. The entire assay was repeated four independent times. The assay was quantified using phase contrast microscopy to obtain images for analysis. Three representative images (at 10× magnification) were captured for each sample. These images were quantified by Adobe Illustrator 2023 and Adobe Photoshop 2023, followed by ImageJ analysis by three independent observers in a randomized double-blind manner. The data is graphed using GraphPad Prism (Version 9). All data are plotted as mean ± standard deviation. Data were analyzed through an unpaired non-parametric *t*-test followed by the Mann-Whitney test. All analyses should be completed using a 95% confidence interval. Data is considered significant when *P* < 0.05.

*Note: Our laboratory uses Adobe Illustrator and Adobe Photoshop combined with ImageJ to perform the analysis of data obtained in the MDA. We realize that the Adobe Illustrator and Adobe Photoshop software are not readily available to all laboratories. Alternative programs for data analysis include open-source image processing programs like ImageJ or Fiji. These platforms offer a vast array of tools and macros, such as the Drawing Tools set available at this GitHub location:* *https://github.com/fiji/fiji/blob/master/macros/toolsets/Drawing%20Tools.txt*. *Such open-source alternatives would broaden the protocol's accessibility, benefiting a larger number of users. However, we have no experience in using these open-source software programs, so we cannot comment on their efficacy for data analysis.*

## Validation of protocol

This MDA protocol was used in the following research papers:

- Dom, A.M., Buckley, A.W., Brown, K.C., Egleton, R.D., Marcelo, A.J., Proper, N.A., Weller, D.E., Shah, Y.H., Lau, J.K., Dasgupta, P. (2011) The α7-nicotinic acetylcholine receptor and MMP-2/-9 pathway mediate the proangiogenic effect of nicotine in human retinal endothelial cells. *Invest. Ophthalmol. Vis. Sci.*, 52, 4428-4438.

## General notes and troubleshooting

**Troubleshooting tips**

1. HRMECs should be cultured to approximately 70%–80% confluence. Replace culture medium at least once in three days. Avoid growing the cells to more than 80% confluence to prevent contact inhibition and senescence of cultures.
2. It is very important that the HRMECs are healthy for the assay. Cells should display a cobblestone morphology and non‐granular cytoplasm. Therefore, we usually use HRMECs between passage 3 and 7 for all our experiments.
3. The HRMECs should be carefully trypsinized. Excessive trypsinization (or insufficient neutralization of trypsin) promotes cell aggregation and cell death and releases cellular debris, which will compromise the quality of the assay.
4. The Matrigel aliquot should be thawed overnight at 4 °C. Alternately, the Matrigel aliquot may be thawed on ice (for 5–6 h) before performing the experiment. Avoid rapid thawing of the Matrigel.
5. When working with Matrigel, use pre-chilled pipette tips. Alternatively, fill a 15 mL centrifuge tube with ice-cold sterile PBS or base media to cool the tip by filling and ejecting the cold liquid several times immediately before use with the Matrigel.
6. It is very important to aliquot the Matrigel. Do not re-freeze Matrigel more than twice. Repeated freeze and thaw of the Matrigel stock or aliquots will degrade the basement membrane proteins within the Matrigel. Similarly, storage of Matrigel at room temperature will cause it to polymerize (solidify), rendering it unusable.
7. Keep the mix of HRMECs and Matrigel (for the primary layer) on ice to prevent the solidification of Matrigel. Make sure to pipette up and down to keep the cells uniformly suspended before plating. Do not vortex the mixture of HRMECs and Matrigel. Perform gentle pipetting to avoid forming any bubbles in the Matrigel during the process.
8. The secondary layer comprises a 1:1 solution of phenol red–free Matrigel diluted in EGM-2 supplemented with 4% FBS. Mix the phenol red–free Matrigel and EGM-2 (supplemented with 4% FBS) by gently pipetting up and down. Avoid agitating the tubes or vortexing them as this will create air bubbles in the secondary layer.

## Supplementary information

**Raw data for the MDA obtained from ImageJ(CTRL-M) function**

Each sample was tested in duplicate in the MDA. The entire assay was repeated four independent times. The assay was quantified using phase contrast microscopy to obtain images for analysis. Three representative images(at 10× magnification) were captured for each sample. These images were quantified by Adobe Illustrator 2023 and Adobe Photoshop 2023 followed by ImageJ analysis by three independent observers in a randomized double-blind manner.

1. The terms Control-1, Control-2, and Control-3(in EXCELOBSERVER-1-MDA-COUNTS-RAW-DATA, indicated by the green colored rows R1, R2, R3) represent three images(of three independent fields) of the vehicle-treated HRMECs. Similarly, PP2-10 µM-1, PP2-10 µM-2, and PP2-10 µM-3, indicated by orange-colored rows R4, R5, R6(EXCELOBSERVER-1-MDA-COUNTS-RAW-DATA), represent three images(of three independent fields) of PP2-treated HRMECs. A similar annotation has been used for EXCELOBSERVER-2-MDA-COUNTS-RAW-DATA and EXCELOBSERVER-3-MDA-COUNTS-RAW-DATA.
2. In the file EXCELOBSERVER-1-MDA-COUNTS-RAW-DATA, columns C1–C6 represent the dimensions of the six lines drawn for each image(analyzed by ImageJ followed by CRTL-M), as described by OBSERVER-1. The column C7 marked as“AVERAGE”(and indicated in yellow) represents the average of each row. The numbers in column C9(in pink color) show the average from the first three numbers(rows R1, R2, R3) in column C7. This number is the average of all vehicle-treated control HRMECs(indicated by pink color and referred as VEH in column C8) for Experiment-1. This process was repeated for Experiments 2-4. A similar annotation has been used for EXCELOBSERVER-2-MDA-COUNTS-RAW-DATA and EXCELOBSERVER-3-MDA-COUNTS-RAW-DATA.
3. The numbers in column C8 and row R5(in blue color) show the average of numbers in the last three rows(R4, R5, R6) of the column C7. This number is the average of the PP2-treated HRMECs(indicated by blue color and referred as PP2-Av in column C8) for Experiment-1. This process was repeated for Experiments 2-4. A similar annotation has been used for EXCELOBSERVER-2-MDA-COUNTS-RAW-DATA and EXCELOBSERVER-3-MDA-COUNTS-RAW-DATA.
4. The above-mentioned process yielded 12 values(from three observers over four experiments) for vehicle-treated control HRMECs, which are represented in Column A(yellow column) in the file EXCELDATA-PROCESSING-FROM-THREE-OBSERVERS. Similarly, the 12 values for PP2-treated HRMECs are shown in Column B(red column) in the file EXCELDATA-PROCESSING-FROM-THREE-OBSERVERS. The average of the numbers for vehicle-treated control(Column A) was calculated(= 595.5). Using this value, data was converted into percentage of control(Table 4, Columns C and D). The numbers in columns C and D were used to generate the graph(Figure 11) in GraphPad Prism.

The following supporting information can be downloaded here:

1. EXCELSUPPL-DATA-OBSERVER-1-MDA-COUNTS-RAW-DATA: Raw data obtained by first observer.
2. EXCELSUPPL-DATA-OBSERVER-2-MDA-COUNTS-RAW-DATA: Raw data obtained by second observer.
3. EXCELSUPPL-DATA-OBSERVER-3-MDA-COUNTS-RAW-DATA: Raw data obtained by third observer.
4. EXCEL SUPPL-DATA-PROCESSING-RESULTS-FROM-THREE-OBSERVERS: Processing the raw data from the observers to create the graph(Figure 11) using GraphPad Prism.
